# Experimental and Analytical Study on the Short-Term Behavior of Locally Bonded Connections in Bamboo–UHPC Composite Beams

**DOI:** 10.3390/ma18061224

**Published:** 2025-03-10

**Authors:** Kang Zhao, Yang Wei, Zicheng Yan, Qiqi Li, Xiayun Fang

**Affiliations:** 1 College of Civil Engineering, Nanjing Forestry University, Nanjing 210037, Chinajysxgzl@163.com (Q.L.); 13585180246@163.com (X.F.); 2 Jiangsu Highway Intelligent Detection and Low-Carbon Maintenance Engineering Research Center, Nanjing Forestry University, Nanjing 210037, China

**Keywords:** bamboo, UHPC, composite beams, bending behavior, locally bonded

## Abstract

The synergistic application of ultra-high-performance concrete (UHPC) and bamboo scrimber provides innovative solutions for sustainable structural engineering. In this study, the structural response mechanism of the combined beams under the steel plate–screw composite connection system was systematically investigated by designing three shear connection gradient specimens (TS200/300/400) to address the key scientific issues of the mechanical behavior of the bamboo–UHPC interface. Based on the unidirectional compression tests of bamboo–UHPC composite shear connections and four-point bending tests of composite beams, the damage modes, load-mid-span deflection relationship, bending stiffness, bamboo–UHPC slip and normal lift were evaluated for all the composite beams with the shear connection gradient as a parameter. The results showed that the flexural performance of the composite beams went through three stages: elastic behavior, damage development and final damage. The interfacial slip and interfacial lift-off have more obvious asymmetric spatial distribution characteristics, and increasing the shear joint degree can delay the separation between the UHPC and the bamboo layer, thus enhancing the structural integrity. Typical features of the final damage are the bending damage of ultra-high-performance concrete and bamboo fiber damage. This study highlights the potential of UHPC–bamboo composite beams for sustainable construction and emphasizes the importance of optimizing shear connection for improved performance.

## 1. Introduction

Unlike other construction materials such as concrete and steel, bamboo is a lightweight and renewable resource. It has a short growth cycle and can be harvested repeatedly, and bamboo waste can also be recycled and processed into new engineering materials [1,2,3]. In addition to its lightweight properties, bamboo also exhibits excellent tensile strength and flexural performance [4,5]. However, as a building material, raw bamboo has limitations such as relatively low stiffness, insufficient durability when exposed to extreme climatic conditions for long periods, and significant susceptibility of mechanical properties to humidity [6,7,8]. To overcome technical bottlenecks such as the size constraints of structural components due to the original bamboo specifications, engineered bamboo has been developed and widely used. A typical example of a modern high-quality material is ultra-high-performance concrete (UHPC), a cement-based composite material known for its exceptional compressive strength, outstanding environmental resistance, and high stiffness. By combining UHPC as a bridge deck material with laminated bamboo main beams to form a laminated bamboo–UHPC composite beam structure, compared to traditional bamboo–concrete composite beam bridges, the self-weight can be reduced by decreasing the thickness, achieving a synergistic development of ecological sustainability and engineering high performance in the structure

Bamboo–concrete composite (BCC) beams represent a novel type of composite component, offering significant advantages over traditional bamboo structures. Native bamboo structures are prone to excessive vibration due to insufficient stiffness, whereas the BCC system enhances the structural damping ratio by approximately two times through composite effects [9,10,11], with flexural stiffness and load-bearing capacity reaching 2.9–4.2 times and 1.2–1.5 times that of pure bamboo structures, respectively [12,13]. The core of the BCC system lies in the reliable connection at the bamboo–concrete interface [14,15,16]. Research on the connection methods between bamboo and concrete has been systematically elaborated in the relevant literature, such as the perforated steel plate interlocking connection [13,16] and the steel mesh embedding connection [17,18,19,20], among other innovative techniques.

Most existing studies focus on the combination of glued-laminated timber (glulam) and ultra-high-performance concrete (UHPC). Replacing ordinary concrete with UHPC can reduce the self-weight by 40–50% [21]. When UHPC is used as a replacement for ordinary concrete, it allows for the construction of lighter, more delicate structures with no reduction in load-bearing capacity, achieving optimization of the ratio between static load and live load [22,23]. The static performance of timber-UHPC composite beams with different spans has been investigated using theoretical analysis and model tests [24,25]. It was found that the thickness of UHPC slabs could be reduced by about 50% to achieve the same load-carrying capacity compared to plain concrete, and the damage mainly occurred at the timber beams and timber–UHPC interfaces. A number of studies [26,27,28] have also been carried out to analyze and compare the effect of the bond stiffness of four different types of bonded joints on the load-carrying capacity, damage modes and slip properties of timber–UHPC interfaces. The stiffness of the adhesive had little overall impact, with interface failure mostly occurring in the wood portion.

As a newly engineered biomass material, bamboo scrimber exhibits significantly superior mechanical properties compared to glued timber. However, when subjected to bending loads, BCC composite beams experience a reduction in overall bending stiffness due to the failure degradation of shear connectors. Previous studies have identified glued connections as less ductile but offering high stiffness, prompting an investigation into the potential of combining localized gluing with screw reinforcement in BCC systems to enhance ductility. This paper proposes a novel bamboo–UHPC composite beam shear connection, incorporating the dual objectives of weight reduction and the implementation of these improvement strategies. Shear connector push-out tests are conducted, and bending performance tests of the composite beams are performed using the degree of shear connection as a parameter. This study analyzes the factors influencing bending capacity and evaluation criteria and explores improvements in load–displacement response prediction methods.

## 2. Materials and Methods

### 2.1. Bamboo

The reconstituted bamboo material used in this experiment was provided by Hongyu Bamboo Industry Co., Ltd., in Xuancheng, Anhui, China. The reconstituted bamboo was produced using a hot-pressing process, which involves splitting the bamboo into 50 mm loose mesh fiber bundles, removing the internal bamboo green and yellow sections, and drying it to reduce the moisture content to below 5%. After carbonization, the internal moisture content of the bamboo fibers is further reduced, while the bamboo fibers are hardened. The bamboo is then impregnated with adhesive, achieving an adhesive uptake of 85%, with a total adhesive uptake of 400 g/m^2^. After drying, the material is formed in a hot-press mold, and after curing, the final density of the reconstituted bamboo reaches 1.32 g/m^3^. To obtain the mechanical properties of the bamboo, ten tension and compression specimens were evaluated. The compression specimen size is 30 mm × 20 mm × 20 mm, the dog bone sample with a tensile specimen has a size of 25 mm × 20 mm × 370 mm, and the specimen size is accurate to 1 mm. Due to the special manufacturing process of hot-pressed bamboo, the internal density after glue-soaking and hot-pressing, the moisture content of bamboo is much lower than that of round bamboo. Therefore, the influence of the moisture content is ignored when analyzing the compressive tensile properties of bamboo in this analysis. Ten compression test specimens and ten tensile test specimens were carried out on bamboo. The ultimate tensile strength and MOE were 125.28 MPa (Cov14.03%) and 15.10 GPa (Cov10.59%), respectively. The ultimate compressive strength and MOE measured were 96.35 MPa (Cov 5%) and 15.43 GPa(Cov 4%), respectively.

### 2.2. Ultra-High-Performance Concrete (UHPC)

The UHPC used in this paper is a commercial premix product, SBT^®^-HDC, and the mixing ratio used is premix/steel fiber/admixture/tap water = 1000 g:20 g:12.5 g:100 g; the admixture product’s name is PCA-I. UHPC is naturally cured and molded in an indoor environment. The compressive strength (fcu) measured with a cube with a side length of 100 mm is 120 MPa, and the compressive strength (fc) and elastic modulus (Ec) measured with a φ100 mm × 200 mm cylinder are 118 MPa and 40.5 GPa, respectively. Its ultimate tensile strength ft is 7.5 MPa and its slump extends to 750 mm.

### 2.3. Shear Parts

**Figure 1:**
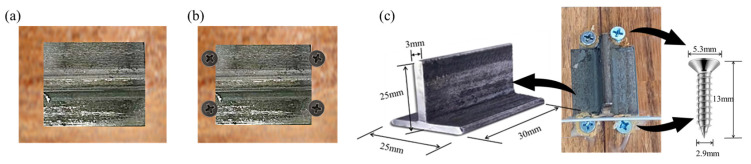
Shear connector; (**a**) T-series; (**b**) TS-series; (**c**) dimensions of each part.

The shear components utilized in this study are illustrated in Figure 1, with dimensions of 25 mm × 25 mm × 3 mm. Each individual shear component measures 30 mm in length and is fabricated from Q235B steel. According to the supplier’s data, the tensile yield strength, the tensile limit strength, and the after-break extension are considered. The lengths are 264.3 MPa, 428.3 MPa and 35%, respectively.

This study involves two types of shear key configurations: the T-series and the TS-series. The T-series consists of three specimens, each featuring a T-shaped steel plate bonded to a bamboo sheet using modified polypropionic acid adhesive, as depicted in Figure 1a. The TS-series also includes three parallel specimens, which build upon the T-series design by incorporating four short stainless steel 304 screws positioned around the steel plate, as illustrated in Figure 1b. Detailed dimensions of each component of the shear keys are provided in Figure 1c.

According to supplier’s data, Young’s modulus for bonding is not lower than 1000 MPa, the tensile strength is not lower than 25 MPa, and the shear strength is not lower than 10 MPa.

## 3. Push-Out Test (Connections)

In composite beams, the shear connection is a critical part for load transfer. The degree of connection of the shear connection directly affects the state of coordinated stresses in composite beams [29,30]; so, it is necessary to carry out the push-out test of the shear connection before the bending test. The push-out test can directly simulate the force behavior of the shear connection in the pure shear state and evaluate its shear load-carrying capacity, slip stiffness and ductility properties. The test can obtain the load–slip curve of the shear connection, determine its ultimate load-carrying capacity, elastic stiffness and damage mode, and provide data support for subsequent testing and evaluation of composite beam forming.

### 3.1. Specimen Preparation and Test Setup (Connections)

**Figure 2:**
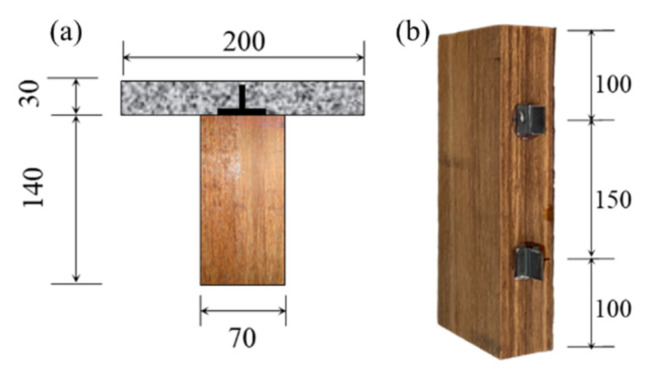
Configurations of the composite beams: (**a**) cross-section; (**b**) shear connection arrangement.

In this experiment, a total of six shear connector specimens divided into two groups were designed for push-out tests. The shear connectors are composed of UHPC panels combined with bamboo, with cross-sectional dimensions as shown in Figure 2a. With reference to previous studies [12,13,31], each shear connector measures 350 mm in length and is spaced longitudinally at 150 mm intervals on the laminated bamboo, as depicted in Figure 2b. The T-series shear connectors consist of three specimens (with steel plates bonded to the bamboo using modified polyacrylate adhesive resin), while the TS-series shear connectors include three parallel specimens (based on the T-series, with four short screws arranged around the steel plates).

**Figure 3:**
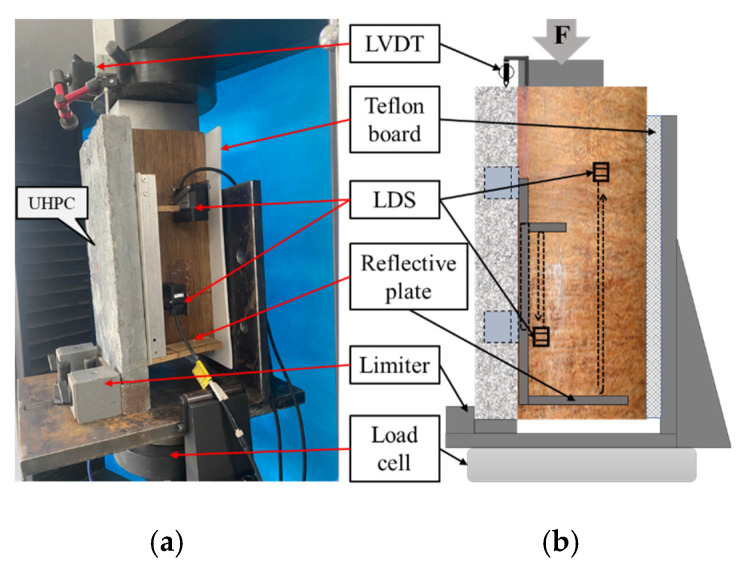
Test setup of the push-out tests: (**a**) actual; (**b**) schematic.

The push-out test setup is illustrated in Figure 3, employing a unidirectional shear configuration. This unidirectional shear configuration is made of high-strength steel; the size of the base plate is 35 cm × 35 cm, the size of the side baffle is 35 cm × 35 cm, and the back of the side baffle is reinforced with triangular steel plates, all of which are 2 cm thick and connected by welding.

The UHPC portion is placed on a limiter, while the laminated bamboo section remains suspended. Polytetrafluoroethylene (PTFE) plates are utilized on the sides to minimize frictional resistance caused by lateral contact [32]. A load cell is positioned beneath the fixture, and one LVDT (Linear Variable Differential Transformer) is placed at the top of the specimen. Additionally, two laser displacement sensors are symmetrically arranged at the shear connector locations on the bamboo section, resulting in a total of five displacement measurement devices to determine the average displacement. All push-out specimens are subjected to monotonic loading. The instrument used for the specimen is a Shenzhen Sens (Shenzhen, China) 100 kN electronic universal testing machine; the displacement test is carried out using the Keyence IL300 model. Prior to the formal test, the specimens are preloaded 10 times between 1 and 5 kN to eliminate any gaps. During the formal loading phase, a monotonic displacement loading is applied at a rate of 0.2 mm/min.

### 3.2. Test Results and Discussion

**Figure 4:**
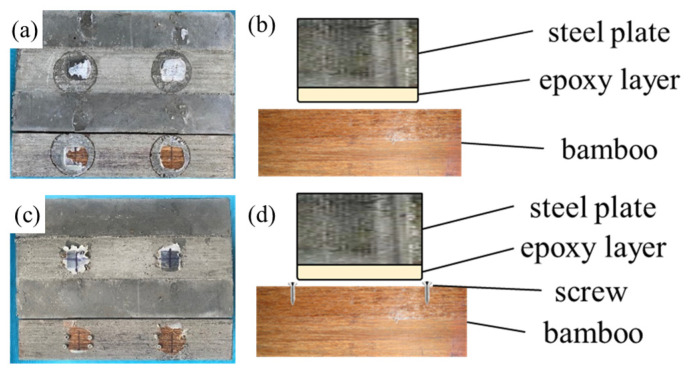
Shear connection failure mode: (**a**) T-Series; (**b**) T-Series failure mode schematic; (**c**) TS-Series; (**d**) TS-Series failure mode schematic.

The failure modes of the shear connectors are depicted in Figure 4, providing a detailed visual representation of the observed behaviors under loading conditions. The most prominent failure mode observed in the experiments is the debonding between the T-shaped steel plates and the laminated bamboo. This debonding failure occurs when the adhesive interface separating the steel plates from the bamboo fails, causing the adhesive to detach from the bamboo surface while remaining bonded to the steel plates. This phenomenon is clearly illustrated in Figure 4a,b, where the separation between the bamboo and the adhesive layer is evident, highlighting the weak interfacial bond as a critical point of failure.

In the TS-series of specimens, an additional observation was made regarding the behavior of the screws used to connect the steel plates to the bamboo. Despite the debonding failure, the screws remained firmly embedded in the bamboo, as shown in Figure 4c,d. This indicates that the mechanical interlocking provided by the screws was effective in maintaining some level of connection even after the adhesive bond had failed. The retention of the screws in the bamboo suggests that the screw–bamboo interface possesses a higher deformability compared to the adhesive–bamboo interface, which failed earlier under the applied loads.

These findings underscore the importance of optimizing both the adhesive bonding and mechanical fastening methods in composite structures to enhance the overall performance and durability of shear connectors. The debonding failure highlights the need for improved adhesive materials or surface treatment techniques to strengthen the bond between steel plates and laminated bamboo. Meanwhile, the effective performance of the screws suggests that mechanical fastening can serve as a reliable secondary mechanism to maintain structural integrity even after adhesive failure. These insights are crucial for the design and development of more robust and resilient composite systems in future applications.

**Figure 5:**
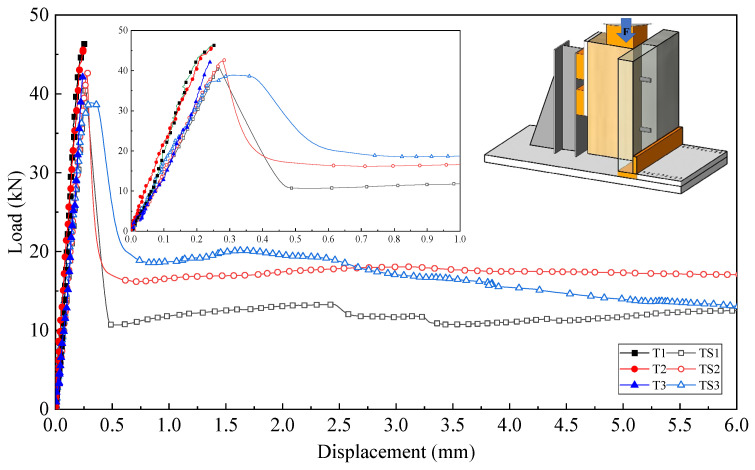
Force–slip curve.

Figure 5 presents the load–slip curves of the shear connectors. In the initial loading phase, both the T and TS groups exhibit an approximately linear relationship between load and displacement, demonstrating high stiffness. Upon reaching the ultimate load, the T-series connectors fail due to debonding at the interface between the laminated bamboo and the modified polyacrylate adhesive, resulting in a loss of connection. For the TS-series, after reaching the peak load, the load rapidly decreases to approximately 40% of the peak value. At this stage, the shear force at the bamboo–UHPC interface is solely carried by the short screws, and the curve exhibits a prolonged plateau. Even at a slip of 10 mm, the load retains about 25% of the peak load-carrying capacity. Subsequently, due to excessive deformation beyond the screw’s deformation capacity, the UHPC panel detaches, leading to the final failure of the specimen.

The key results of the push-out tests for the shear connectors are summarized in Table 1, where K_0.4_ and K_0.8_ represent the shear stiffness values provided by a single dowel at 40% and 80% of the ultimate load, respectively.

## 4. Bending Tests (Beams)

### 4.1. Specimen Preparation and Test Setup (Beams)

**Figure 6:**
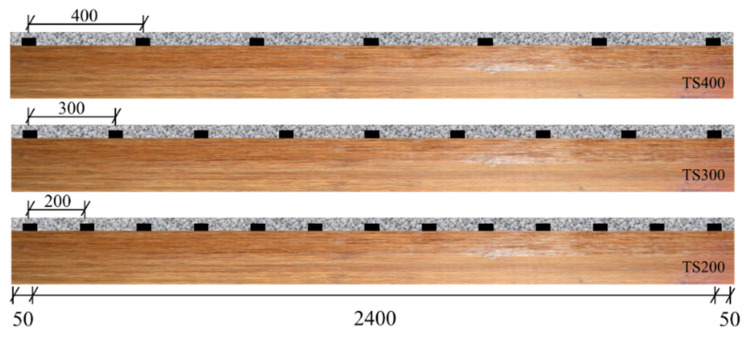
Composite beam layout form.

From the previous experimental section, it can be observed that the peak loads of the T-shaped steel plates combined with short screws are relatively similar. However, the combination ensures a certain load-bearing capacity after the peak load is reached (approximately 40% to 50% of the peak load). To investigate the influence of different connection methods on the flexural performance of composite beams, based on the results of the shear connector push-out tests, three full-scale composite beams were fabricated using the TS-series shear connectors. Along with a pure bamboo beam, a total of four beam specimens were subjected to flexural performance testing. The composite beams are composed of laminated bamboo and UHPC connected together, with the cross-section of the composite beams being identical to that of the shear push-out specimens. The composite beams are 2500 mm in length with a support span of 2400 mm. The UHPC portion has a height of 35 mm and a width of 200 mm, while the laminated bamboo has a height of 140 mm and a width of 70 mm. The experimental setup includes a reference beam B0, and three beams with varying shear connector spacings designated as TS200, TS300, and TS400, with spacings of 200 mm, 300 mm, and 400 mm, respectively (as shown in Figure 6).

During loading, the test beams were subjected to a four-point bending configuration, with both the reference beam and the composite beams positioned centrally. The span between the supports was set at 2400 mm. The test beams were divided into three equal segments of 800 mm by the loading points. A 100 kN servo actuator was employed as the loading device, and the test was conducted under displacement control at a loading rate of 2 mm/min. Prior to the formal loading, each specimen was preloaded 15 times with a force of 10 kN to ensure the elimination of any gaps, after which the loading continued until the specimen failed.

**Figure 7:**
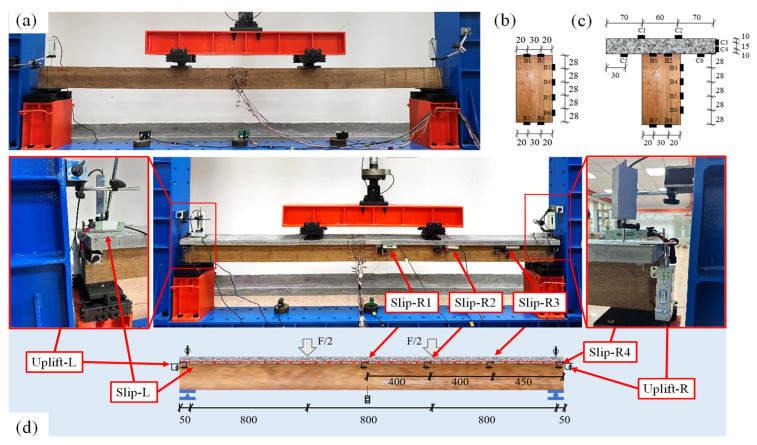
Arrangements of instrumentation and loading system: (**a**) B0 beam; (**b**) strain gauges location of B0; (**c**) strain gauges location of composite beam; (**d**) composite beam.

The loading schematic of the test beams is illustrated in Figure 7. For the pure bamboo beam, measurements were taken for the mid-span and support displacements (as shown in Figure 7a) and the mid-span cross-sectional strains. Strain gauges were arranged as depicted in Figure 7b, with two strain gauges placed on both the top and bottom surfaces of the beam, and four strain gauges evenly spaced along the side of the bamboo beam. For the composite beams, the following data were measured: (1) displacements at the mid-span and support locations of the composite beams, as shown in Figure 7c; (2) mid-span cross-sectional strains, with strain gauges arranged as shown in Figure 7c, where the bamboo beam’s strain gauge arrangement was identical to that of the pure bamboo beam, and two strain gauges were placed on both the top and bottom surfaces as well as the sides of the UHPC section; (3) relative slip between the UHPC and the bamboo beam at the interface, with slip measurement points set at intervals of 400 mm, 400 mm, and 450 mm from the mid-span to the right end of the composite beam, designated as Slip-R1, Slip-R2, Slip-R3, and Slip-R4, respectively, and an additional slip measurement point at the left end of the beam, named Slip-L; (4) uplift displacement between the UHPC and the laminated bamboo beam, with uplift displacement measurement points located at both ends of the composite beam, designated as Uplift-L and Uplift-R. Displacement and strain data were collected using the TDS-540 data acquisition system produced by the Tokyo Measuring Instruments Laboratory.

### 4.2. Failure Modes

In the initial loading phase, the composite beams exhibited a linear and continuous deflection under the applied load, with no visible signs of damage or significant phenomena. This phase demonstrated the elastic behavior of the beams, where the UHPC and bamboo components worked in unison to resist the load effectively. However, as the load increased, the composite beams began to exhibit signs of distress, marking the transition to the progressive damage phase. During this stage, adhesive layer peeling became noticeable, particularly at the interface between the UHPC and bamboo layers, accompanied by audible cracking sounds emanating from the screw connection points. These sounds were indicative of localized failures in the UHPC and the gradual breakdown of the bond between the materials.

With further loading, the frequency of these failure sounds increased, and the nonlinear behavior of the composite beams became more pronounced. The slip between the UHPC and bamboo layers, as well as the uplift effect at the ends of the beams, intensified, signaling a reduction in the composite action and a redistribution of stresses within the structure. As the load approached the ultimate capacity, a loud noise was heard, marking the onset of complete failure. At this point, significant uplift was observed at both ends of the UHPC panels, indicating the loss of composite action and the separation of the UHPC from the bamboo beams. Simultaneously, the bamboo, which had been subjected to increasing tensile strain at the bottom, reached its maximum strain capacity and fractured, leading to the rupture of the bamboo beams.

**Figure 8:**
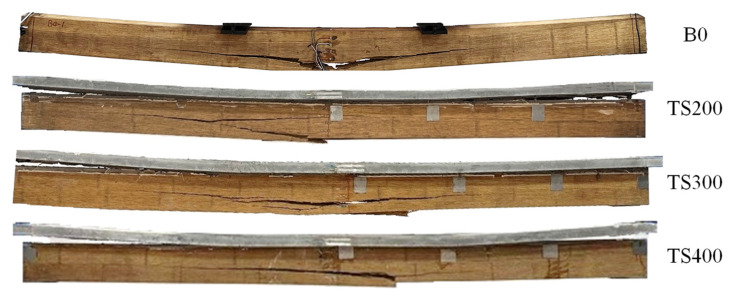
Failure mode of composite beams.

All test beams ultimately failed due to the fracture of bamboo fibers, which acted as the primary tensile reinforcement. This failure was accompanied by the uplift and peeling of the UHPC panels at both ends of the composite beams, as well as transverse cracking in the UHPC panels. These transverse cracks were likely caused by the bending stresses and the loss of bond between the UHPC and bamboo layers. The failure modes of each beam, as illustrated in Figure 8, provide a clear visual representation of the progressive damage and ultimate failure mechanisms, highlighting the critical role of the bamboo–UHPC interface and the importance of optimizing the shear connection to enhance the overall performance of such composite systems. These findings underscore the need for further research into improving the bond strength and load transfer mechanisms in UHPC–bamboo composite beams to achieve greater structural efficiency and durability.

**Figure 9:**
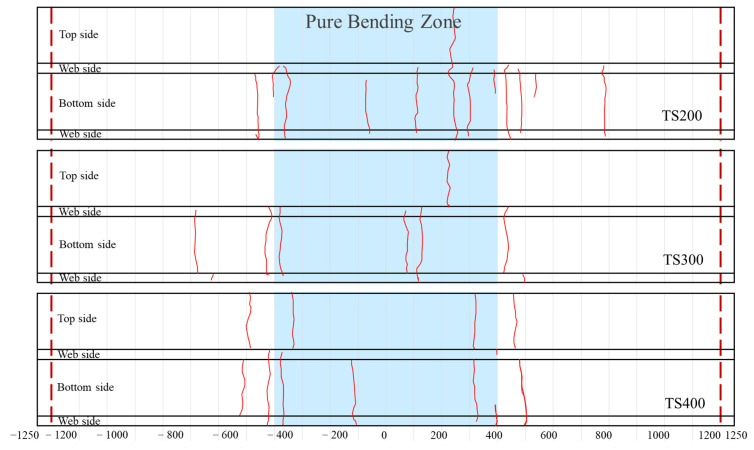
Distribution diagram of fractures of composite beams.

The crack distribution is illustrated in Figure 9, which shows the crack patterns on the top, bottom, and side webs of each beam. The red dashed lines indicate the support points, and the pure bending regions of the composite beams are highlighted in blue. The cracks on the UHPC are highlighted with a solid red line. As can be seen from the figure, the cracks in the UHPC panels are all transverse. The number of transverse cracks in the base plate of TS200, TS300, and TS400 specimens were 12, 6, and 6, respectively. Comparing TS200, TS300, and TS400, as the number of shear connectors increases, the number of transverse cracks in the UHPC bottom panels also increases. Moreover, the cracks predominantly appear in the pure bending regions of the composite beams. The bending stress dominant zone (mid-span ± L/4) collects 50% to 58% of the transverse cracks, and their average spacing increases from 133 mm at TS200 to 200 mm at TS400. The transverse cracks on the top panels are distributed around the loading points, where the cracks are likely due to localized stress concentrations at the contact areas.

### 4.3. Load–Displacement Curves

**Figure 10:**
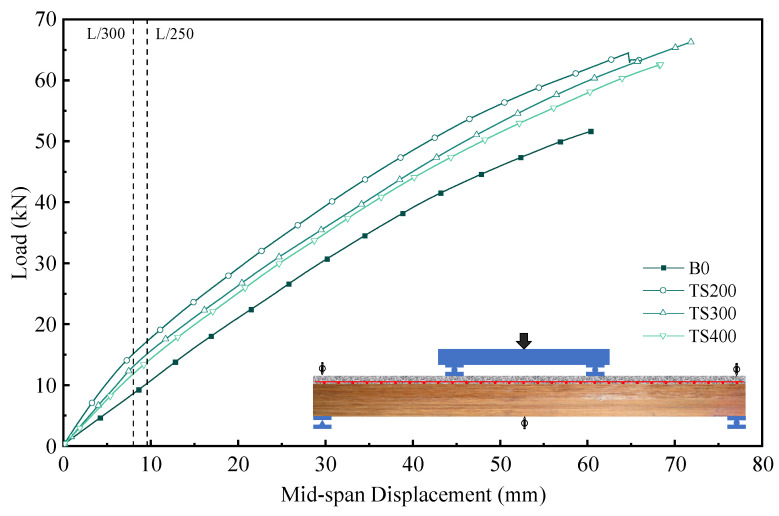
Load displacement curve of composite beam midspan.

According to the Chinese timber structure code and Eurocode 5, the deflection limits for timber structures under serviceability limit states are specified as L/250 and L/300, respectively. As shown in Table 2, when the connector spacing was reduced from 400 mm to 200 mm (increasing the number of connectors from 7 to 13), the load at L/300 deflection (P_L/300_) for the composite beams increased from 11.86 kN to 15 kN, representing a significant improvement of 37% to 73% compared to the original beams. Similarly, the stiffness at L/300 deflection (EI_L/300_) for the composite beams with connector spacings of 400 mm, 300 mm, and 200 mm was 1.38, 1.52, and 1.76 times that of the original beams, respectively. This study found that increasing the number of connectors effectively enhances the stiffness of the composite beams. However, when the mid-span deflection reached L/250, the load at L/250 (P_L/250_) increased by 35% to 67% compared to the original beams, and the stiffness at L/250 (EI_L/250_) was 1.34, 1.47, and 1.67 times that of the original beams, respectively. Compared to the results at L/300, the increase in load-bearing capacity and stiffness at L/250 was reduced, which is attributed to the failure of the connectors. Notably as shown in Figure 10, the stiffness of the original beams remained unchanged at both L/300 and L/250, indicating that they were still in the elastic stage. This, combined with the experimental observations, further confirms that the performance degradation of the composite beams is primarily due to the interface between UHPC and laminated bamboo. Additionally, the ultimate load-bearing capacity of the composite beams also showed improvement compared to the original beams. When the connector spacing was reduced from 400 mm to 200 mm, the ultimate load-bearing capacity of the composite beams increased by 21% to 28% compared to the original beams.

### 4.4. Strain Distribution of Each Beam

**Figure 11:**
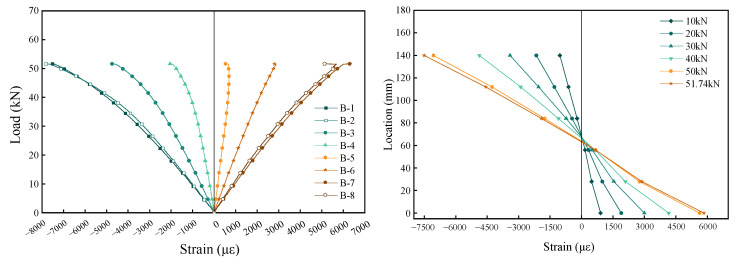
Bamboo beam B0 medium span section strain.

Figure 11 illustrates the strain distribution of each test beam during the loading process. As can be seen from the figure, the original beam exhibits excellent mechanical performance during loading, with the neutral axis position remaining relatively stable and showing minimal variation. The strain in the beam initially follows a linear relationship during the early stages of loading. However, as the load approaches the ultimate capacity, nonlinear behavior emerges, indicating the development of some plastic deformation. Throughout the loading process, the beam largely adheres to the plane section assumption.

**Figure 12:**
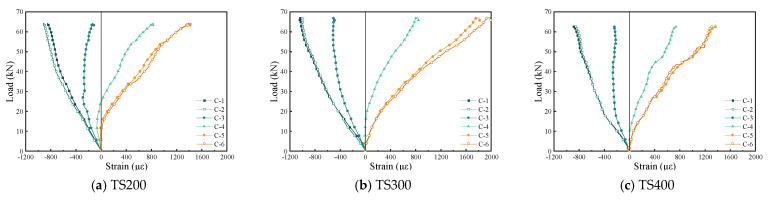
UHPC strain in the middle span section of composite beam.

**Figure 13:**
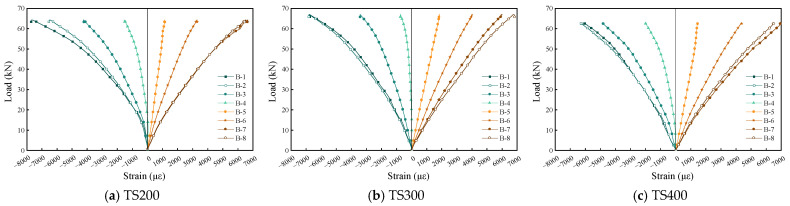
Strain of bamboo beam in the middle span section of composite beam.

**Figure 14:**
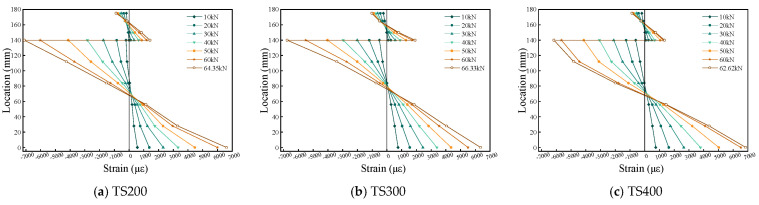
Strain distribution in mid-span section.

Figure 12, Figure 13 and Figure 14 depict the strain development in the concrete, bamboo beam, and mid-span cross-section of the composite beams, respectively. In contrast, the mechanical behavior of the composite beams exhibits significant differences. The composite beams demonstrate an incomplete composite action from the initial loading stage, and the overall behavior does not conform to the plane section assumption. As the load increases, the neutral axis of the composite beams gradually shifts downward, with substantial variation. This phenomenon is primarily due to the significant difference in elastic moduli between the concrete and laminated bamboo, resulting in a noticeable strain discrepancy at the bamboo–concrete interface. Additionally, due to insufficient connection effectiveness, the UHPC panels experience a complex stress state with compression at the top and tension at the bottom during loading. It is noteworthy that the degree of shear connection has a certain influence on the stress distribution in the UHPC. As shown in Figure 12, the concrete section is entirely under compression during the initial loading phase, which aligns with the design intent of the composite beams (ensuring the concrete section is in compression while the tensile stress is borne by the bamboo beam). However, as the load increases and the shear connectors gradually fail, the concrete section begins to experience tension. Taking the strain measurement point C4 at the lower part of the web as an example, TS200 experiences tension at 24.56 kN, TS300 at 13.80 kN, and TS400 at 8.76 kN. Ultimately, the failure mode of the composite beams is characterized by the fracture of the laminated bamboo at the bottom due to reaching the maximum tensile strain. This failure mode further validates the complexity of the mechanical behavior of the composite beams, which arises from the material property differences and insufficient connection effectiveness during loading.

### 4.5. Slip and Uplift in Composite Beams

**Figure 15:**
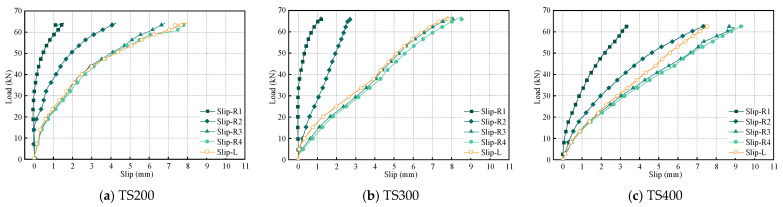
Load slip during composite beam loading.

Figure 15 shows the load–slip curves of each composite beam under different loads. As previously mentioned, the slip measurements are divided into Slip-R1, Slip-R2, Slip-R3, Slip-R4, and Slip-L. R1 and R2 are located in the pure bending region of the composite beam, with R1 at the mid-span and R2 directly below the loading point. R3 is positioned at the three-quarter span, while R4 and L represent the slip at the beam ends.

In the initial loading stage (load level ≤ 10% Pu), the interface shear distribution presents typical elastic characteristics, and the composite beam system exhibits a significant cooperative working mechanism. At this time, the relative slip between the beams was maintained below 0.35 mm, and the load–displacement curves presented an approximate ideal linear elastic response, indicating that the bond interface was in a complete working state and the structural system maintained sufficient combined stiffness. It is worth noting that the slip evolution rate shows a nonlinear acceleration trend when the load increases over 10 kN. As the load continues to increase, the rate of slip accelerates, particularly at the beam ends, where the slip exceeds the peak slip limit, causing the shear connectors to enter a plateau phase and the stiffness of the composite beams to gradually decrease. By comparison, it is observed that the slip values at the two external loading points are larger and similar. Initially, the slip values at both ends of the composite beams are almost identical, indicating a certain degree of specimen uniformity. However, as the load increases and the shear connectors gradually fail, the failure is not symmetrical, leading to an asymmetrical bending response in the composite beams.

Particularly noteworthy is the asymmetric evolution of the mid-span slip value (Slip-R1) revealing a shift in the overall bending response of the composite beams. For TS200 and TS300 with higher shear connections, Slip-R1 starts to show slip values when the load reaches 50% of the peak load, while for TS400 with weaker connections, slip already starts to appear at 20% of the peak load, and the composite beams show an asymmetric bending response, which can also be seen in the slip deviation of Slip-L and Slip-R4.

**Figure 16:**
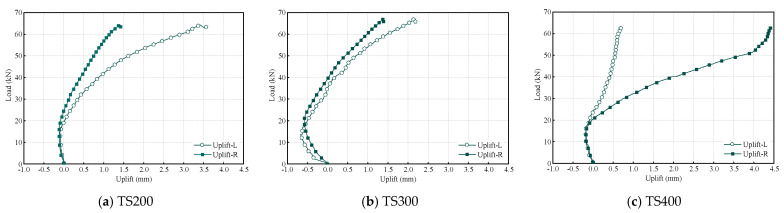
The uplift during composite beam loading.

Figure 16 presents the load–uplift curves for each composite beam. By comparing these curves, it is evident that the uplift values at the left and right ends of the beams vary, but they generally follow a similar trend. During the initial loading phase, due to the presence of gaps between the UHPC and the laminated bamboo, the UHPC section is compressed against the laminated bamboo under the applied force. This corresponds to a mixed phase of interface gap closure and friction locking. When the load reaches approximately 20 kN, the UHPC panel begins to uplift as the connectors debond and fail. The group of shear connectors enters a cascade failure mode, at which point the lift curve exhibits significant nonlinear characteristics: the UHPC panel warps and deforms, resulting in geometric nonlinearity of the composite beam. Geometric nonlinearity of the composite beam due to deformation. The experimental data reveal that the test beams exhibit an asymmetrical uplift response under symmetrical loading, with the maximum uplift reaching 4.4 mm. The displacement difference between the left and right measurement points fluctuates within a range of 0.45 to 3.71 mm. This discrepancy primarily stems from the randomness in the manufacturing of the shear connectors and the anisotropic characteristics of the contact surface roughness.

In practical engineering applications, it is advisable to increase the anchorage length of the connectors within the concrete to enhance the connection performance, thereby ensuring sufficient shear and pull-out resistance.

## 5. Discussion

For timber–concrete composite beams, Eurocode 5 [33] provides a method for calculating the effective flexural stiffness in the elastic stage. In the case of laminated bamboo beams and concrete slabs connected by shear connectors, significant slip still occurs at the interface. Therefore, the effective flexural stiffness is generally calculated using methods similar to those for timber–concrete composite structures, as shown in Equation (1). The relevant parameters in Equation (1) are calculated using Equations (2) and (3). Compared to the ideal fully composite condition, the composite action between laminated bamboo and concrete beams should actually be defined as “partial composite action”. The flexural stiffness calculations for laminated bamboo–concrete composite beams under the two extreme conditions of fully composite action and no-composite action are given by Equations (5) and (6), respectively. The relationship between displacement and load in the elastic stage is described by Equation (7).

(1) $$EI_{\mathrm{eff}}=E_{U}I_{U}+E_{b}I_{b}+\gamma E_{U}A_{U}Z_{U}^{2}+E_{b}A_{b}Z_{b}^{2}$$

(2) $$\gamma =\frac{1}{1+\frac{\pi^{2}E_{U}A_{U}s_{s}}{K_{s}l^{2}}}$$

(3) $$Z_{b}=\frac{\gamma E_{U}A_{U}(h_{U}+h_{b})}{2(\gamma E_{U}A_{U}+E_{b}A_{b})}$$

(4) $$Z_{U}=\frac{h_{U}+h_{b}}{2}-Z_{b}$$

(5) $$EI_{\mathrm{full}}=E_{U}I_{U}+E_{b}I_{b}+E_{U}A_{U}Z_{U}^{2}+E_{b}A_{b}Z_{b}^{2}$$

(6) $$EI_{\mathrm{non}}=E_{U}I_{U}+E_{b}I_{b}$$

(7) $$\Delta =\frac{Pa}{48EI_{eff}}(3l^{2}-4a^{2})$$

In the equations, the subscripts U and b represent UHPC and bamboo, respectively; E, I, A, and h denote the elastic modulus, moment of inertia, cross-sectional area, and section height, respectively; s_s_ and K_s_ represent the spacing and slip stiffness of the shear connectors, respectively. Here, the calculated stiffness uses K.

**Figure 17:**
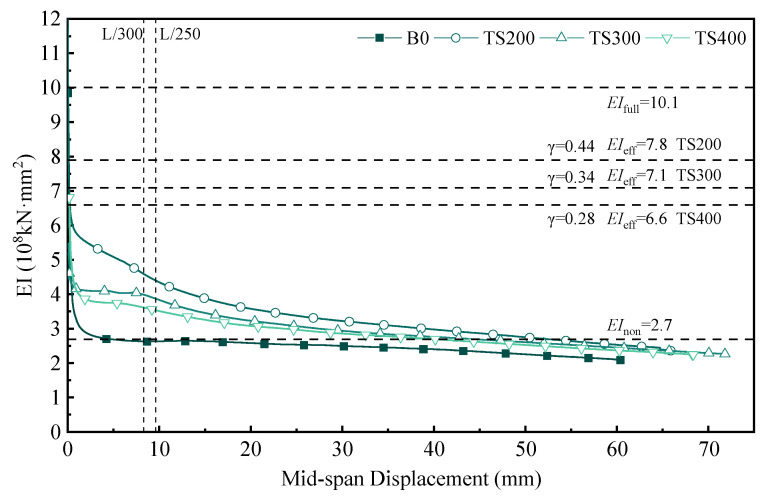
Bending stiffness–displacement relationship curve of composite beam.

To more intuitively compare the variation in flexural stiffness of each test beam during the bending process, the relationship between flexural stiffness and mid-span displacement is plotted, as shown in Figure 17.

During the bending loading process, the flexural stiffness of each test beam exhibits a decreasing trend. The stiffness is highest at the initial loading stage, but as the load increases, the shear connectors gradually degrade, and the composite effect of the beams weakens, leading to a reduction in stiffness. Before the mid-span displacement reaches L/300, the composite beams are in a partially composite state. As shown in Table 1, when the mid-span displacement reaches L/300, the flexural moduli of the composite beams are 0.59 (TS200), 0.56 (TS300), and 0.55 (TS400), respectively. Beyond the L/300 displacement threshold, the rate of stiffness reduction slows down, approaching the non-composite state. It should be noted that the stiffness values in the figure are calculated using Equation (7), which assumes the beam is in the elastic stage. Therefore, as shown in Figure 17, when the mid-span displacement reaches a certain level (Δ ≥ 40 mm), the stiffness values are close to the non-composite state, and in the later stages of the test, they even fall below the non-composite state. This is because the beam enters the plastic stage in the later phase of the test, making Equation (7) no longer applicable. However, the formula remains valid in the early loading stage when the beam is in the elastic stage.

In order to quantitatively study the combined effect of shear joints on wood–concrete combination beams, Gustawski et al. [34] proposed the concept of the combination coefficient DCA; Δ_NC_ is the theoretical deflection of wood–concrete combination beams without connection; Δ_PC_ is the experimental deflection value of the wood–concrete combination beam; Δ_FC_ is the theoretical deflection of the wood–concrete combination beam without interface slip.

(8) $$DCA=\frac{\Delta_{NC}-\Delta_{PC}}{\Delta_{NC}-\Delta_{FC}}$$

**Figure 18:**
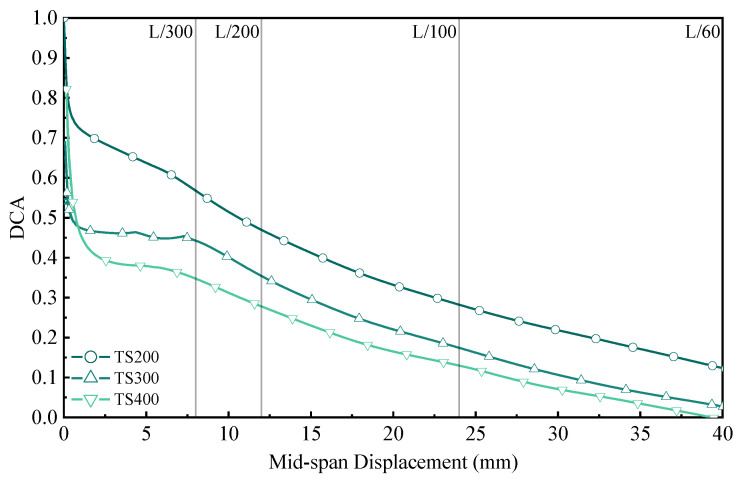
Combination coefficient–displacement curve of composite beam.

For the fully combined structure DCA = 1; for the non-combination structure DCA = 0; the larger the combined coefficient DCA, the higher the degree of combination, and the greater the structural stiffness and bearing capacity. According to the above results, only the cross-median displacement was analyzed for this experiment. The variation pattern of combination coefficients within 40 mm is shown in Figure 18. Overall, the change trend of the combination coefficient of the combined beam is consistent with the bending stiffness in Figure 16. At the beginning of loading, the subsequent curve suddenly fell. When the span displacement value reached L/300, the combination coefficients were only 0.58, 0.45, and 0.36. The combination coefficient of subsequent combination beams continued to decline until about Δ to 40 mm (L/60); the combination coefficient was close to the no-combination state. The combination beams entered the plastic stage due to material damage and deterioration of UHPC and bamboo, etc.; in particular, UHPC cracks could no longer be calculated based on the full-length span.

The experimental bamboo–concrete combination beams in this paper are calculated according to Equation (5) to Equation (6). When γ = 0 is taken, the calculation result is that there is no combination of the combined beam. When γ = 1.0 is taken, the calculation result is a complete combination of rigid connections of the combined beam. The EI_eff_ is calculated based on the shear slip stiffness K_0.4_. The research teams in previous studies found that the elastic bending stiffness of bamboo–concrete composite beams requires a reduction coefficient (0.6~0.8) based on the γ method [12,13,16,31]. For the combined beams proposed in this paper, *EI*_eff_ is reduced by 0.6 according to the research above. The load displacement response of the combined beam in L/300 can be well predicted.

From Figure 17 and Figure 18, it can be concluded that the combined beam is close to the no-combination state outside L/300. Here, *EI*_composite_ is proposed. When Δ ≤ L/300, 0.6EI_eff_ is used for calculation, and when Δ > L/300, *EI*_non_ is used for calculation.

(9) $$EI_{composite}=\left\{\begin{array}{ll} 0.6EI_{eff} & \Delta \leq L/300\\ EI_{non} & \Delta >L/300 \end{array}\right.$$

**Figure 19:**
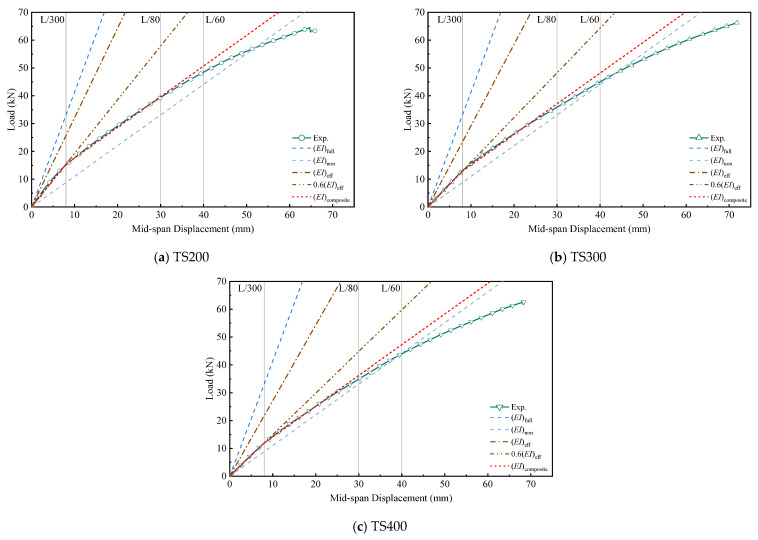
Comparison of the theoretical curve of load–displacement and test curve of composite beam.

Figure 19 is the experimental value of the load displacement of each test beam and the prediction curve of different stiffness values. Through the test results, it can be seen that the test curve in the range of 0~L/60 is between the full combination curve and the no-combination curve.

When Δ ≤ L/300, the 0.6EI_eff_ prediction curve and the test curve overlap higher. When Δ > L/300, the 0.6EIeff prediction curve gradually deviates from the experimental curve. The composite calculated stiffness EI_composite_ proposed in this paper is in the range of L/80. The test load displacement curve can be predicted better. It is worth noting that the degree of shear connection of the combined beam is still an important factor in determining the bending performance of the combined beam. For this article, using EI_composite_ when Δ ≤ L/80 can be used to predict load displacement more closely, when Δ > L/80, the prediction curve gradually deviates from the experimental value, and the higher the degree of shear connection, the later the deviation. This is because in L/80, the combined beams are in the combined elastic state and the separated elastic state, respectively. The load continues to increase, and as the degree of shear transmission failure becomes higher, each part of the combined beam bears the bending load until each part enters the plastic deformation range, until the bamboo beam finally breaks and breaks.

## 6. Conclusions

This paper focuses on the bending performance of locally bonded reconstituted bamboo–UHPC composite beams. The bending capacity and stiffness variation patterns were analyzed with the degree of shear connection as a parameter, and a bilinear load–displacement response curve was proposed. The main conclusions are as follows:

(1) Both adhesive-only and adhesive–screw composite shear connectors exhibited a linear load–slip relationship before reaching peak load in push-out tests. Adhesive-only shear connectors showed brittle failure, while adhesive–screw composite connectors retained 40% of their load-bearing capacity after peak load, demonstrating better ductility.
(2) For the adhesive–screw composite beams studied in this paper, the bending performance tests revealed three typical stages: two elastic stages with different stiffnesses and one plastic stage. Overall, the bending behavior of the composite beams exhibited nonlinear flexural characteristics.
(3) The traditional γ-method is not entirely applicable to the novel composite beams proposed in this study. Introducing a reduction factor provided a reasonable prediction of the load–displacement response within the L/300 range. The bilinear stiffness calculation method offered a more accurate prediction of the load–displacement response within the L/80 range. And the higher the shear connectivity, the later the timing of the deviation of the experimental curves from the predicted curves. However, due to the limited number of test samples in this study, further validation is required. For example, further research on the effect of parameters such as the length of the screw implanted bamboo and the thickness of the UHPC board on the composite beam is needed.

## Figures and Tables

**Table 1:** Shear connections launch experiment results.

	Ultimate Load (kN)	Slip at Ultimate Load (mm)	K_0.4_ (kN/mm)	Ave K_0.4_ (kN/mm)	COV (%)	K_0.8_ (kN/mm)	Ave K_0.8_ (kN/mm)	COV (%)
T1	46.35	0.25	96.52	100.61	10.00	101.30	95.44	8.96
T2	45.57	0.24	114.46			101.68		
T3	42.12	0.23	90.84			83.36		
TS1	41.08	0.26	75.23	77.09	2.00	72.16	74.13	2.12
TS2	42.63	0.28	79.01			74.24		
TS3	38.88	0.30	77.02			76.00		

**Table 2:** UHPC–bamboo composite beam test results.

Specimen	P_L/300_	EI_L/300_	P_L/250_	EI_L/250_	Pu
B0	8.66	2.63	10.29	2.63	51.74
TS200	15.00	4.65	17.23	4.40	63.35
TS300	13.17	4.00	15.21	3.87	66.28
TS400	11.86	3.63	13.85	3.53	62.58

## Data Availability

The original contributions presented in this study are included in the article. Further inquiries can be directed to the corresponding authors.
